# The Neuroinflammatory Etiopathology of Myalgic Encephalomyelitis/Chronic Fatigue Syndrome (ME/CFS)

**DOI:** 10.3389/fphys.2017.00088

**Published:** 2017-02-17

**Authors:** Julian A. G. Glassford

**Affiliations:** Independent Health Researcher and ConsultantShrewsbury, UK

**Keywords:** chronic neurotrophic infection, glial hyper-activation, ME/CFS, neural sensitization, neuroimmune, neuroinflammation, neurotoxic neuroexcitation, noxious nociception

## Abstract

Myalgic Encephalomyelitis/Chronic Fatigue Syndrome (ME/CFS) is a debilitating multi-systemic chronic illness of unknown etiology, classified as a neurological disorder by the World Health Organization (WHO). The symptomatology of the condition appears to emanate from a variety of sources of chronic neurological disturbance and associated distortions, and chronicity, in noxious sensory signaling and neuroimmune activation. This article incorporates a summary review and discussion of biomedical research considered relevant to this essential conception perspective. It is intended to provide stakeholders with a concise, integrated outline disease model in order to help demystify this major public health problem. The primary etiopathological factors presented are: (A) Postural/biomechanical pain signaling, affecting adverse neuroexcitation, in the context of compression, constriction, strain, or damage of vertebral-regional bone and neuromuscular tissues; (B) Immune mediated inflammatory sequelae, in the context of prolonged immunotropic neurotrophic infection—with lymphotropic/gliotropic/glio-toxic varieties implicated in particular; (C) A combination of factors A and B. Sustained glial activation under such conditions is associated with oxidative and nitrosative stress, neuroinflammation, and neural sensitivity. These processes collectively enhance the potential for multi-systemic disarray involving endocrine pathway aberration, immune and mitochondrial dysfunction, and neurodegeneration, and tend toward still more intractable synergistic neuro-glial dysfunction (gliopathy), autoimmunity, and central neuronal sensitization.

## Introduction

Myalgic Encephalomyelitis/Chronic Fatigue Syndrome (ME/CFS) is an idiopathic, heterogeneous condition involving distortion of homeostasis across multiple organ systems. It is chiefly characterized by myalgia, fatigue, neurocognitive dysfunction, and, critically, delayed muscle recovery (Paul et al., 1999) and the intensification of symptoms following physical exertion: “Post Exertional Malaise” (PEM), or “Post Exertional Neuroimmune Exhaustion” (PENE) (VanNess et al., 2010).

This highly intrusive disease involves varying degrees of physical disability and cognitive deficits, associated psychosocial difficulties, and significantly reduced quality of life (Winger et al., 2015) and life expectancy (Jason et al., 2006), across a patient population numbering in the millions worldwide. Complete/spontaneous recovery is extremely rare and conventional treatment strategies rarely deliver even modest direct, objective and, sustained symptomatic improvement. Thus, ME/CFS constitutes a particularly enigmatic, debilitating, and costly major public health issue, and the advancement of our understanding of its essence, hence, a pressing area of biomedical enquiry (Arroll, 2014).

The central focus of this work is the proposition that ME/CFS constitutes the symptomatic manifestation of enhanced nervous sensitivity attributable to a neuroinflammatory etiopathology associated with abnormal nociceptive and neuroimmune activity. The article reviews, highlights, and interconnects numerous relevant disease features, processes, and concepts from the biomedical literature, the ultimate aim of which is to provide stakeholders with an instructive pathophysiological conceptual framework.

## Discussion

### Neuroanatomical input

Postural and biomechanically induced neuroexcitatory neuroinflammation has been posited as a partial explanation for PEM/PENE (Rowe et al., 2013), and sustained muscle activity and associated cortical excitability have been observed in ME/CFS (Brouwer and Packer, 1994).

A number of clinical and physical therapy researchers have, over the course of recent decades, variously formed associations between ME/CFS, orthostatic intolerance, and Ehlers-Danlos syndrome (Rowe et al., 1999), ME/CFS and joint hypermobility (Barron et al., 2002), and ME/CFS and innate (flat thoracic spine), osteochondritis, or trauma-linked vertebral defects (Perrin, 2007). These issues, along with other connective tissue disorders, fractures, and other factors that reduce range of motion (ROM), a physical impediment common among ME/CFS patients (Rowe et al., 2014), plus lengthy periods of reduced movement of neuromuscular tissues, may be associated with diminished neural motility and increased neuromuscular tension/strain. Both during and 24 h following strain-inducing experimental physical maneuvers, ME/CFS patients report significantly greater symptom intensity elevation than do sham exposed patient and healthy control cohorts (Rowe et al., 2016). Along with neuropathies and peri-neural adhesions, these issues represent potential ongoing sources of nociceptive input and glial activation, resulting in enhanced peripheral and central nervous sensitivity (Rowe et al., 2013). Peri-neural adhesions are fibrous bonds proximal to nerve tissues, often formed during post-operative/injury healing.

Any residual central sensitivity may, in turn, effect heightened sensitivity of relevant portions of the peripheral nervous system (PNS) to further input (Rowe et al., 2014). Furthermore, neurons may continue to fire after the initiating stimulation has ceased (Löscher and Ebert, 1996): a phenomenon observed in relation to persistent, relatively low threshold stimulation, colloquially referred to as “kindling” (Jason et al., 2011). Thus, the potential exists for the emergence of an insidious peripheral-central neurogenic sensitization loop, which would conceivably have the power to modulate the impact of symptom worsening events in ME/CFS. Hypothesized pathological products of these processes include increased resting muscle tone, affected by enhanced tension in/stimulation from proximal peri-neural fibers subject to sensitization, and aggravation of vascular and autonomic tone (Rowe et al., 2013).

### Glial activation

Nociceptive afferent input excites post-synaptic neurons and may also be read by glia, triggering cellular responses e.g., via the stimulated neuronal release of chemical mediators that bind to glial receptors (Ren and Dubner, 2008). Calcium ion (Ca(2+)) influx into astrocytes following stimulation causes central terminals of the nociceptor to release a host of neuroactive signal molecules. These include the primary neuroexcitatory neurotransmitter, glutamate, nitric oxide (NO), and pro-inflammatory cytokines: Tumor necrosis factor alpha (TNF-α) and interleukin-1 beta (IL-1β) (Ricci et al., 2009).

Activated microglia behave similarly in responding to immune challenge/inflammation (Renno et al., 1995), also inducing superoxide production. Superoxide and NO are free radical substrates of the potent, toxic oxidant peroxynitrite (ONOO–), and hence sources of oxidative and nitrosative (O+NS) damage, both individually and, particularly, when combined (Barger et al., 2007). Along with the abovementioned stimuli, glia may also be primed to respond more harshly by exposure to toxins and electromagnetic fields (EMF), including non-ionizing, radio frequency (RFR) electromagnetic radiation (EMR) (Hao et al., 2010), autoimmune processes (Colton, 2009), and the effects of aging (Norden and Godbout, 2012).

Glia propagate inflammatory signals and may cause chronic pain e.g., involving allodynia and hyperalgesia (Yasui et al., 2014), via the impact of bidirectional neuro-glial signaling; glia may be activated by way of neuronal stimulation (as described above) and the inflammatory cytokines that they release may, in turn, couple to neuronal glutamate receptors, thereby enhancing neuroexcitation (Ren and Dubner, 2008). Synergistic interactions may result, prompting the dysregulation of glial functions (gliopathy) and associated maladaptive hyper-activation (microgliosis) (Ji et al., 2013). Magnetic resonance imaging (MRI) reveals changes in the structure of the brain in ME/CFS that may indeed be attributable to astrocyte dysfunction (Barnden et al., 2011).

Gliopathy can come about following exposure to “glio-toxins”: Immunosuppressive mycotoxins produced by certain species of fungi, including house mold (Aikawa and Suzuki, 1985). In one study, 93% of ME/CFS patients tested positive for mycotoxins (Brewer et al., 2013), compared with 0% of controls at similar detection thresholds (Hooper et al., 2009).^*^ Gliopathies are also believed to be initiated, more broadly, by concurrent increases in extracellular glutamate and cytokine production (Hulsebosch, 2008).

Enhanced glial activation results in neuroexcitation, neuroinflammation and neurodegeneration, indicated by significantly reduced white and gray matter in ME/CFS (Puri et al., 2012), and hence (additional) glial multiplication subject to neuronal damage; at the symptomatic level it is associated with cognitive deficits, common in ME/CFS (Shanks et al., 2013), and depressive-like behavior (Norden and Godbout, 2012), also fairly common in the condition (Maes and Twisk, 2010). Widespread low-level neuroinflammation has been detected in the brains of ME/CFS patients, with neuropsychological symptom severity associated with the level of neuroinflammation in one study (Nakatomi et al., 2014), and with self-reported chronic pain in another (Ickmans et al., 2015). Furthermore, animal testing appears to indicate that microglial induction of IL-1β may be a prerequisite for immunologically induced fatigue (Ifuku et al., 2014),^**^ and that gender-specific relationships may exist concerning suppression of T cell contact-mediated glial activation (Brahmachari and Pahan, 2010) and relative prolongation of glial responses following neurotoxin exposure (Ciesielska et al., 2009). These findings accord with numerous human studies highlighting relatively high prevalence of ME/CFS among females (Reyes et al., 2003).

^*^ Elevated levels of pro-inflammatory cytokines paired with O+NS stress are suspected as being behind mitochondrial dysfunction in the disease (Morris and Maes, 2012). Associated depletion of antioxidants may account for some of the damage to fatty acids, proteins, and mitochondrial DNA and membranes associated with ME/CFS, via the activity of reactive oxygen and nitrogen species (ROS/RNS) (Maes and Twisk, 2010). The anomalous presence of mycotoxins may play a role here too (Brewer et al., 2013), and EMR exposure represents a complicating factor within this thematic e.g., given its capacity to influence fungal activity/biotoxicity (Velizarov et al., 1999) and lipid peroxidation (Mailankot et al., 2009).
^**^ IL-1β mediates substances that may induce excitotoxicity (Mohebiany and Schneider, 2013), and inflammatory cytokine activity could play an important role in the aberration of endocrine (Jason et al., 2011) and mitochondrial dysfunction (Myhill et al., 2009).

### Central sensitivity

Peripheral pain can contribute to “central sensitization,” or “wind-up”: The enhancement of nociceptive pathways in the central nervous system (CNS), a process arguably manifest in hyperalgesia in ME/CFS (Nijs et al., 2012) and sister condition Fibromyalgia Syndrome (FMS) (Woolf, 2011), and one which has been used to theoretically elucidate aspects of ME/CFS symptomatology (Meeus and Nijs, 2007). Wind-up takes place under long-term potentiation (LTP), in the context of temporal summation effected by repetitive nociceptive stimulation (which may be low/subclinical in intensity) (Woolf, 1991).

ME/CFS patients have demonstrably low nociceptive thresholds of the muscle tissues (Vecchiet et al., 1996), and many have significant cerebral (brain) hypoperfusion (Hamre, 1995), including in the parahippocampus (Gay et al., 2016) and brain stem (Costa et al., 1995), which may contribute to abnormal function of the locus ceruleus, involved in the control of descending inhibitory nociceptive pathways (Meeus and Nijs, 2007). Along with NO and pro-inflammatory cytokine activity, excitotoxicity may play a role in migraine pathogenesis (Longoni and Ferrarese, 2006). Migraines have been detected in 82% of ME/CFS cases and factors implicated in migraine pathology likely contribute to the type of headaches and cognitive impairments seen in the disease. Consistent with the above, the cortical spreading depression (CSD) model of migraine describes dysfunction in brain energetics, the brainstem, and thalamocortical tracts associated with changes in cerebrovascular dynamics and ischemia affecting loss of descending inhibitory nociceptive processes (Rayhan et al., 2013).

### Aberration of endocrine pathways

Pro-inflammatory cytokines can alter the metabolism of serotonin (5-HT) and dopamine (Felger and Miller, 2012),^*^ effecting dysregulation of associated neurotransmitters, including glutamate, norepinephrine (NE, noradrenalin), and corticosteroids. 5-HT and NE are the major neurotransmitters involved in descending nociceptive modulation, and NE increases blood flow/pressure by increasing vascular tone, triggers glucose release, and can suppress neuroinflammation. Glucocorticoid deficiencies^**^ are associated with interrupted nociceptive modulation, for example via the disruption of neuroendocrine-mediated glial activation mechanisms (Schwartzman, 2012).

^*^ Serotonin status fluctuation (Badawy et al., 2005) and dopaminergic modulation abnormalities (Georgiades et al., 2003) have been observed in ME/CFS.
^**^ Both increased resistance to glucocorticoid-immune signaling (Kavelaars et al., 2000) and increased glucocorticoid receptor sensitivity have been implicated in ME/CFS (Visser et al., 2001).

Adenosine triphosphate (ATP) activity influences glutamate and gamma-Aminobutyric acid (GABA) release, and hence the glutamate:GABA ratio. GABA is the primary neuroinhibitory neurotransmitter in the CNS. ATP depletion is observed in ME/CFS (Morris and Maes, 2013), and is associated with N-methyl-D-aspartate (NMDA) stimulation and hypersensitivity (Pall, 2003). Furthermore, low neuronal energy production markedly increases sensitivity to glutamate excitotoxicity (Blaylock and Maroon, 2012). NMDA receptor activation results in increased levels of ONOO– (Pall, 2003) and triggers loss of mitochondrial membrane potential and apoptosis (Hardingham et al., 2002).

### Circulatory abnormalities

ME/CFS patients tend to have low cardiac output and the vast majority experience orthostatic intolerance (OI) (Miwa, 2015), a manifestation of dysautonomia relating to inadequate blood circulation and attributable to hypovolemia/blood pooling in the extremities. Various regulatory neurotransmitter/(neuro)endocrine abnormalities (Pall, 2007), as well as (related) sympathetic nervous system (SNS) dominance (Freeman and Komaroff, 1997), and diminished cardiac mass (Hollingsworth et al., 2012), may account for such features. Additionally, cerebral vascular control appears closely related to skeletal muscle pH, found to be elevated in ME/CFS at rest (Jones et al., 2010), and it has been hypothesized that compromised skeletal muscle cellular membrane function may lead to a degree of acidity equalization between the skeletal muscle intracellular environment (raised pH) and the blood (lowered pH). Consistent with these relations, abnormally prolonged cerebral vasoconstriction following orthostatic challenge is routinely observed in ME/CFS (He et al., 2013); this has ramifications for neural health, as per the links outlined in the previous section, plus other deleterious effects including cerebral hypoxia and neurocognitive deficits (Ocon, 2013).

### Gene activity

General statistical surveys are suggestive of a heritable aspect to ME/CFS, with the relative risk of disease development among first degree relatives almost 3 times the norm (Albright et al., 2011). It is worth noting that inflammatory reactivity is of course a function of genetic variation in cytokine genotypes, which underpin the severity of “sickness behavior” in ME/CFS (Vollmer-Conna et al., 2008) e.g., in the context of chronic neurotropic infection (VanElzakker, 2013).

Polymorphism in the TNF-α promoter gene *TNF-857* and significant decrease of *interferon-gamma* (IFNγ) *low producers* have been observed in ME/CFS (Carlo-Stella et al., 2006), implying an inherent pro-inflammatory immunomodulatory disposition. Patients have also been found to have polymorphisms of transient receptor potential channels (TRPs): TRPM1 and TRPM3 (Marshall-Gradisnik et al., 2015), affecting mechanoreceptor nociceptive and mood-linked neurological status, and appear to have peculiar genes and gene activity relating more specifically to aspects of central nociception and stress mediation (CDC, 2006). Abnormally high expression of P2X purinoceptor 7 protein encoding *P2RX7*, a gene that regulates nociception (and inflammatory pain in particular), has been noted (Light et al., 2013) and there is also evidence of an array of serotonergic genetic abnormalities; these include differences in genes responsible for 5-HT production (Narita et al., 2003) as well as distinct genotypic and allelic frequencies of the 5-HT receptor 2A (5-HT2A) encoding *HTR2A* gene (Bozzinia et al., 2012). 5-HT2A receptors are believed to mediate neuronal excitation and anxiety, their expression appears to be up-regulated post-infection (Couch et al., 2015), and their antagonists have been shown to mitigate chronic pain (Bardin, 2011).

### Evolution of encumbrance disorders

The term “encumbrance” is used in this article to denote the constriction, strain, or damage of neuromuscular tissues, as well as associated compression of proximal osseous tissues.

Core intensive/high neuro-dynamic intensity physical activity increases the risk of encumbrance-linked health problems, particularly during spinal development. Related risk factors also include acute neuromuscular strains, neuropathies,^*^ and peri-neural adhesions, as well as relatively indirect processes such as the evolution of connective tissue, hypermobility, and inflammatory disorders. Such issues may be underpinned by genetic vulnerability concerning/the epigenetic impact of psychological trauma (Heim et al., 2009) and psychosocial stress (Prins et al., 2008), together with a broader array of neurotoxic (Giordano and Costa, 2012)/immunotoxic stressors (Dietert, 2014), on the development and functioning of (neuro)anatomic, (neuro)endocrine, and (neuro)immune systems.

^*^ Animal testing reveals that peripheral nerve injury may alter blood-spinal cord barrier (BSCB) integrity (Echeverry et al., 2011), and viruses, inflammatory cytokines, RFR EMR, ONOO–, O+NS stress, and psychological stress may similarly compromise the blood-brain barrier (BBB) (Bested et al., 2001). This renders the central nervous system (CNS) relatively vulnerable to the permeation of pollutants from the blood.

Common postural risk factors associated with constriction/biomechanical strain of neuromuscular tissues subject to abnormal tension and related sensitivity may include: (1) Flexion of the hip or ankle beyond 70 degrees; (2) Flexion of the neck/arching of the back associated with tucking of the chin and forward/stooped head positions; (3) Lower back slumping/jamming/immobilization e.g., in the absence of sufficient ischial support (Rowe et al., 2016). Factor 3 is a potential source of decreased lumbar lordosis, and constriction/compression of proximal dorsal root ganglia (DRG) and osseous tissues, and hence of (enhanced) nociceptive stimulation (Mörl and Bradl, 2013).

Relative inactivity may also have ramifications for the health of neuromuscular tissues, for example via heightened nociceptive/neuroinflammatory responses in the context of enhanced neuromuscular tension/strain resulting from diminished neurodynamic and neuromuscular motility/flexibility (Rowe et al., 2013).

Chronic sleep deficiencies in ME/CFS influence insulin-like growth factor 1 (IGF-1) synthesis, neural sensitivity (Schuh-Hofer et al., 2013), and neurotoxin clearance (Xie et al., 2013). IGF-1 is a hormone stimulated by growth hormone, particularly during deeper “slow-wave” sleep; ME/CFS patients typically experience little sleep at stages III and IV (Fischler et al., 1997) and have lower serum levels of IGF-1 (Berwaerts et al., 1998), with quality of sleep correlated with circulating levels of pro-inflammatory cytokines, and severity and frequency of symptoms, in the disease (Milrad et al., 2017). Additionally, pro-inflammatory cytokines appear to directly dampen IGF-1 pathways (Puche and Castilla-Cortázar, 2012). IGF-1 plays an important role in many biological processes, notably including: Myelination (Liang et al., 2007) and early recovery from demyelination (Mason et al., 2000), and mitochondrial nutrient and inhibitory neurotransmitter synthesis.

### Neuroinflammatory etiopathogenesis

**Figure 1:**
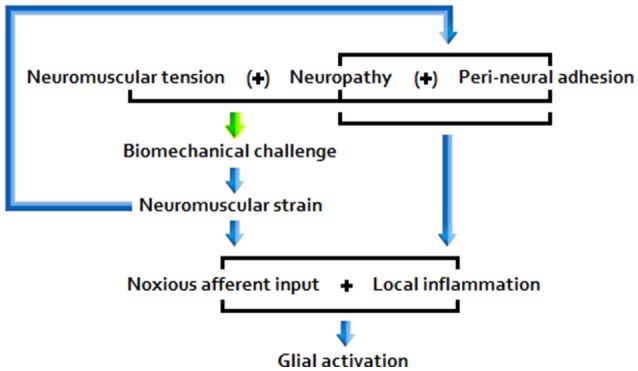
**Encumbrance pathway “A”**.

The picture that emerges from the literature indicates that disease pathogenesis is a function of the following primary etiopathologies: (A) Chronic peripheral nociception/neuroinflammation associated with encumbrance of neuromuscular/osseous tissues (Figure 1); (B) Chronic immune activation associated with marked antigenic activity; (C) A combination of factors A and B.

- Under postural/biomechanical challenge, sensitive neuromuscular tissues subject to encumbrance trigger noxious nociceptive input and glial activity. Responding glial cells propagate inflammatory signals, releasing pro-inflammatory cytokines and effecting central nociceptor terminal release of neuroactive molecules, including glutamate and NO.
- Persistent neuroimmune stimulation, above systemic tolerance thresholds, associated with inadequate immunological responses to neurotrophic infection, often involving lymphotropic/gliotropic microorganisms (Hickie et al., 2006), may lead to immune suppression/exhaustion. In the context of immunological functional impairment tending toward autoimmunity (Bradley et al., 2013), this may involve somewhat circular processes of initial infection, inflammation, paired with enhanced serotonin receptor expression (Couch et al., 2015), and hence raised (inflammatory) pain, immunodeficiency, and progression/reactivation of opportunistic intercurrent (Smith and Thomas, 2015) and latent infections (Broderick et al., 2010); well represented among these pathogens (Nicolson et al., 2003) are those known to target sites of autoimmune inflammation (Posnett and Yarilin, 2005). Consistent with this picture, recurrent viral infections and concordant chronic systemic inflammation appear to be a hallmark of the disease (Raison et al., 2009).
- Neuromuscular tissue inflaming anatomical stimuli modulate local immune responses. Equally, immune stimulation affecting altered serotonergic receptor activity, glial activation, and inflammation of neuromuscular tissues proximal to infection sites also enhances sensitivity of said tissues to postural/biomechanical stimulation. Neural excitation threshold reduction affected by select pathogenic strains may also play a role here (Oldstone, 1989).

**Figure 2:**
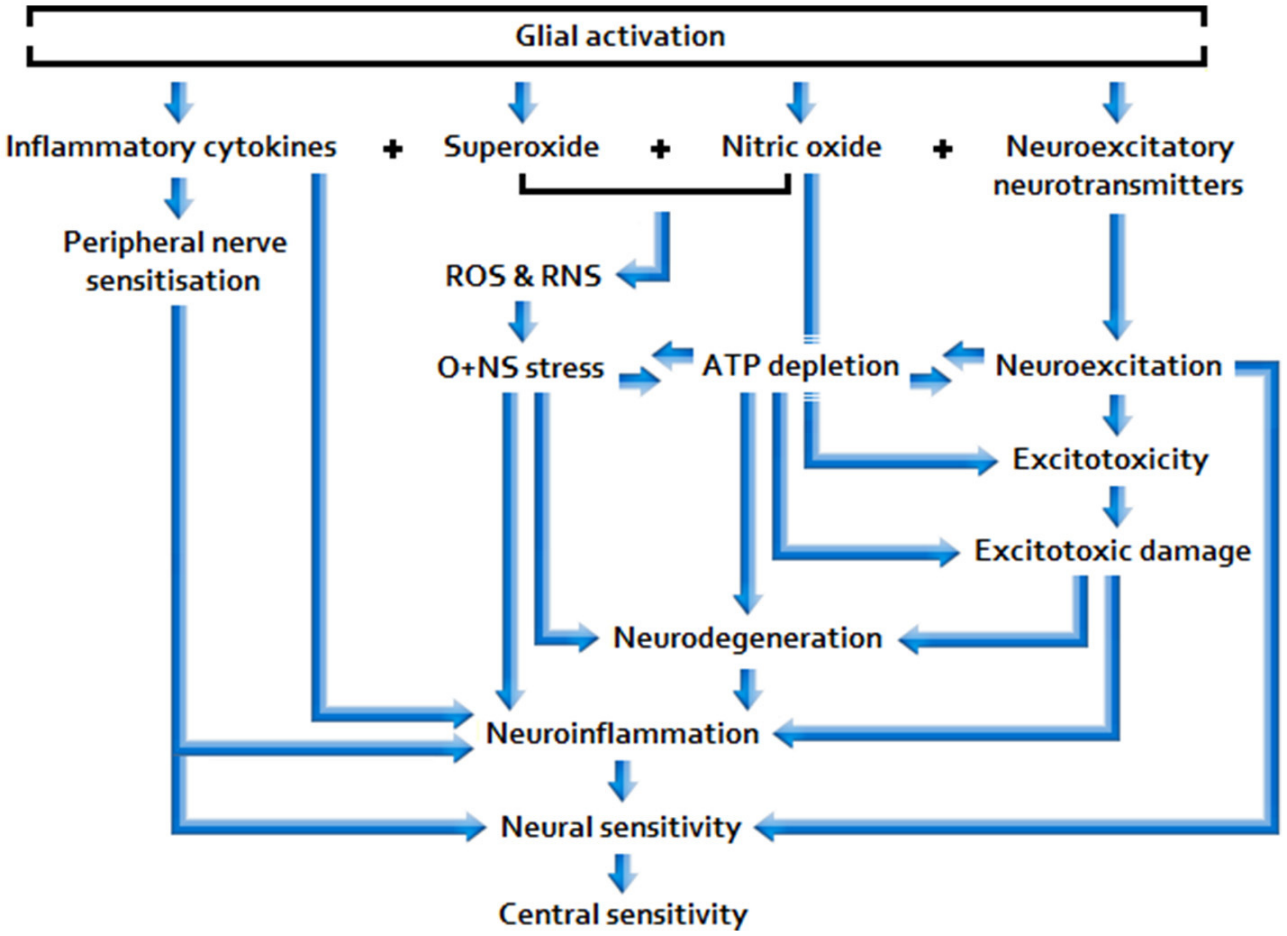
**Effects of sustained glial activation**.

Sufficiently acute/sustained exposure to glio-toxins (B, C) and/or extracellular glutamate and pro-inflammatory cytokine elevation stemming from other neuroinflammatory stimuli (A, B, C) is associated with elevated, and potentially increasingly primed, glial activity and sickness behavior, as well as neuroinflammtory (excitotoxic) neurotoxicity and O+NS stress, and raised peripheral nervous sensitivity. Signal molecules released in relation to these effects act as to lower the threshold and opening characteristics of neuronal receptor channels, hence enhancing central neuronal sensitivity. See Figure 2 for a basic model of adverse products of glial activation and their downstream effects.

Sustained glial activation enhances the potential for longer lasting, more intractable multi-systemic disarray, including space for: Synergistic neuro-glial distortions (i.e., gliopathy/microgliosis), neuroendocrine pathway aberration, mitochondrial dysfunction, and immunodeficiency; hence, glial-simulative neurological activity may have the capacity to fundamentally determine disease severity and prolongation. Given that low-dose Naltrexone (LDN) inhibits glial activation, the reported efficacy of this drug in ME/CFS and FMS would appear consistent with this proposition (Younger et al., 2013).

### Mitigation of encumbrance symptoms

Optimal management/treatment of the encumbrance-linked abnormalities outlined in this article and the supporting literature warrants further study (Rowe et al., 2014). At a minimum, avoiding the noted “common postural risk factors” (flexion/slumping) and intense/repetitive neuro-dynamic movement of sensitive neuromuscular tissues is advisable. The use of special furniture/accessories may aid relevant behavior change e.g., rocking kneeling chairs/wobble cushions and vertebral support belts/braces.

Manual physical therapy focused on mild functional exercises and gentle stretching, intended to gradually increase ROM and reduce neuromuscular tension, may prove beneficial. Physical therapists should be aware, however, that the DRG may be sensitive/inflamed and that manipulation of this region, as well as direct nerve mobilization (particularly in the direction of strain), has the capacity to exacerbate symptoms considerably (Rowe et al., 2013).

Overcoming physical activity avoidance and facilitating physical rehabilitation are considered important recovery goals (Nijs et al., 2013). If progress here is to be optimally sustained, then related endeavors will, thus, remain within dynamic, individualized parameters, reflecting: Levels of strength and stamina (Eyskens et al., 2015), peripheral sensitivity (Staud et al., 2015), limited energy envelopes (Jason et al., 2009), and delayed mitochondrial energy (Lengert and Drossel, 2015) and muscular recovery (Paul et al., 1999).

Complementary dietary supplementation regimens might include: Omega-3 PUFAs (Maes and Twisk, 2010), CoQ10 (Chang et al., 2012), *Withania Somnifera* (*Ashwagandha*, Indian Ginseng) (Sankar et al., 2007), N-acetylcysteine (NAC) (Dean et al., 2011),^*^ vitamin B12 (Zoccolella et al., 2009), curcumin (contained in turmeric) (Blaylock and Maroon, 2012), zinc (Hambidge and Krebs, 2007), magnesium (Cox et al., 1991), 2-aminoethanesulfonic acid (L-Taurine) (Leon et al., 2009), and carnitine (L-Carnitine) (Maes and Twisk, 2010). These substances reportedly aid healthy modulation of glutamatergic, neurotropic, and other relevant inflammatory pathways, protection against/the reversal of excitotoxicity, the rebalancing of glutamate:GABA, and mitochondrial and brain function.

A precautionary approach may be considered sensible with respect to additional factors that could conceivably complicate pathophysiological processes outlined in this article. These may include: Exposure to toxicants e.g., chemical pesticides (Dietert, 2014)/heavy metals (Giordano and Costa, 2012), mold (Aikawa and Suzuki, 1985), and both low frequency (ELF) (Rowland et al., 1998) and high frequency RFR (Pall, 2016) EMFs/EMR, as well as psychological stress (Prins et al., 2008), dehydration (Rowe et al., 1999), and exhaustion (Blaylock and Maroon, 2012).

## Author contributions

The author confirms being the sole contributor of this work and approved it for publication.

### Conflict of interest statement

The author declares that the research was conducted in the absence of any commercial or financial relationships that could be construed as a potential conflict of interest.
